# Acidosis Activates Endoplasmic Reticulum Stress Pathways through GPR4 in Human Vascular Endothelial Cells

**DOI:** 10.3390/ijms18020278

**Published:** 2017-01-27

**Authors:** Lixue Dong, Elizabeth A. Krewson, Li V. Yang

**Affiliations:** 1 Department of Internal Medicine, Brody School of Medicine, East Carolina University, Greenville, NC 27834, USA; dongl14@students.ecu.edu; 2 Department of Anatomy and Cell Biology, Brody School of Medicine, East Carolina University, Greenville, NC 27834, USA; krewsone12@students.ecu.edu

**Keywords:** acidosis, pH, tissue microenvironment, G protein-coupled receptor 4 (GPR4), endothelial cell (EC), blood vessel, endoplasmic reticulum (ER) stress, unfolded protein response (UPR)

## Abstract

Acidosis commonly exists in the tissue microenvironment of various pathophysiological conditions such as tumors, inflammation, ischemia, metabolic disease, and respiratory disease. For instance, the tumor microenvironment is characterized by acidosis and hypoxia due to tumor heterogeneity, aerobic glycolysis (the “Warburg effect”), and the defective vasculature that cannot efficiently deliver oxygen and nutrients or remove metabolic acid byproduct. How the acidic microenvironment affects the function of blood vessels, however, is not well defined. GPR4 (G protein-coupled receptor 4) is a member of the proton-sensing G protein-coupled receptors and it has high expression in endothelial cells (ECs). We have previously reported that acidosis induces a broad inflammatory response in ECs. Acidosis also increases the expression of several endoplasmic reticulum (ER) stress response genes such as CHOP (C/EBP homologous protein) and ATF3 (activating transcription factor 3). In the current study, we have examined acidosis/GPR4-induced ER stress pathways in human umbilical vein endothelial cells (HUVEC) and other types of ECs. All three arms of the ER stress/unfolded protein response (UPR) pathways were activated by acidosis in ECs as an increased expression of phosphorylated eIF2α (eukaryotic initiation factor 2α), phosphorylated IRE1α (inositol-requiring enzyme 1α), and cleaved ATF6 upon acidic pH treatment was observed. The expression of other downstream mediators of the UPR, such as ATF4, ATF3, and spliced XBP-1 (X box-binding protein 1), was also induced by acidosis. Through genetic and pharmacological approaches to modulate the expression level or activity of GPR4 in HUVEC, we found that GPR4 plays an important role in mediating the ER stress response induced by acidosis. As ER stress/UPR can cause inflammation and cell apoptosis, acidosis/GPR4-induced ER stress pathways in ECs may regulate vascular growth and inflammatory response in the acidic microenvironment.

## 1. Introduction

A diligent regulation of pH homeostasis is essential for normal physiological function. Under many pathological conditions, however, proton overproduction and accumulation can cause acidosis in the tissue microenvironment [1,2,3,4,5]. Solid tumors, for example, cancer cells, preferentially utilize aerobic glycolysis (the “Warburg effect”), producing a large amount of lactic acid. This process is further augmented by tumor hypoxia. Moreover, the acid products cannot be efficiently removed due to defective tumor blood vessels [3,4]. As a result, the tumor microenvironment is characteristically acidic, especially in the poorly perfused regions [3,4]. In addition to solid tumors, acidosis also exists in ischemic and inflammatory tissues, in which glycolytic metabolism, hypoxia, and poor blood perfusion lead to proton accumulation and microenvironment acidification [2,5,6,7]. A pH ranging from 6.0 to 7.0 is commonly found in the microenvironment of solid tumors, ischemic tissues, and inflammatory tissues [6,7,8]. Acidosis is a stressor for cells and has been implicated in a variety of cellular processes such as apoptosis, inflammation, angiogenesis, and immune cell response [1,2,3,4,5,6,7].

In addition, acidosis has been demonstrated to stimulate endoplasmic reticulum (ER) stress and unfolded protein response (UPR) in several cell types such as astrocytes [9], mesothelial cells [10], cancer cells [11], and vascular endothelial cells (ECs) [2,12]. However, the molecular mechanisms by which cells sense and respond to acidosis to regulate ER stress response are poorly understood. The ER organelle consists of a continuous tubular network, which functions in the folding and transportation of protein molecules. Imbalance between the demand and aptitude of the protein-folding organelle institutes the UPR/ER-stress response [13,14,15]. Specifically, UPR is mediated through three pathways via the activation of three ER transmembrane sensors, including PERK (protein kinase R-like ER kinase), ATF6 (activating transcription factor 6), and IRE1 (inositol-requiring enzyme 1) [13,14,15]. PERK activation leads to the phosphorylation of eukaryotic initiation factor 2α (eIF2α), which halts protein translation in order to reduce the capacity of protein transportation to the ER. Phosphorylated eIF2α also activates ATF4 that is subsequently directed into the nucleus to upregulate ER chaperones, such as C/EBP homologous protein (CHOP), and induce downstream targets, such as ATF3 [15,16]. Secondly, ATF6 translocates to the Golgi apparatus, where it is cleaved to the activated form p50, which is then translocated to the nucleus to regulate the gene expression of ER chaperones. The third sensor, IRE1, is responsible for the cleavage of X box-binding protein 1 (XBP-1) mRNA to its spliced isoform that encodes a transcription factor regulating multiple UPR genes, such as ER chaperones, and endoplasmic reticulum associated protein degradation (ERAD) [13,14,15]. Although acidosis has been shown to induce ER stress and UPR [2,9,10,11,12], it is largely unclear how cells sense the acidotic microenvironment to modulate the ER stress response.

Numerous studies have speculated as to the mechanism with which the body perceives, adapts to, and counteracts acidosis [4,5,17,18]. Reports have highlighted GPR4, a proton sensor that becomes activated by extracellular acidic pH [1,2,19,20,21]. GPR4 is predominately expressed in ECs [1,2,22]. Previously, studies have shown that activation of GPR4 by acidosis increases the expression of adhesion molecules, cytokines, chemokines, and several ER-stress response genes including *ATF3* and *CHOP* in ECs [2]. The aim of this study was to further investigate the role of acidosis and GPR4 in the ER stress response of vascular ECs.

In this manuscript, we report that GPR4 is a novel mediator for ER stress in response to acidosis in ECs. GPR4 activation by acidosis stimulates all three arms of the ER stress pathways (PERK, ATF6, and IRE1) in ECs. Notably, the treatment of a small molecule inhibitor of GPR4 reduces the ER-stress response, suggesting that therapeutic targeting of GPR4 may be a useful strategy in the treatment of acidosis-induced ER stress and inflammation [1,2,21,23].

## 2. Results

### 2.1. Acidic pH Activates All Three Arms of the ER Stress/UPR Pathways in Vascular ECs

**Figure 1:**
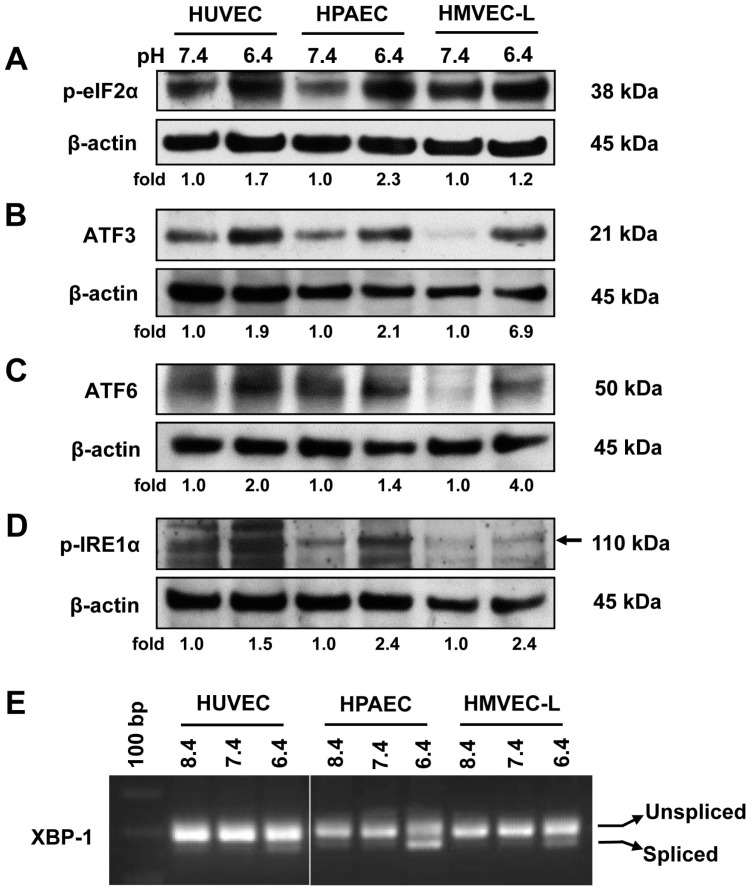
Acidic pH activates the endoplasmic reticulum (ER) stress/unfolded protein response (UPR) pathways in vascular endothelial cells (ECs). (**A**–**D**) Western blot of various protein expression in primary human umbilical vein endothelial cells (HUVEC), human pulmonary artery endothelial cells (HPAEC), and human lung microvascular endothelial cells (HMVEC-L). Cells were treated in EGM-2/HEM or EGM-2-MV/HEM media at physiological (pH 7.4) or acidic (pH 6.4) conditions for 0.5–1 h (p-eIF2α and p-IRE1α), or 5 h (ATF3 and ATF6). Cell lysates were then collected and separated by electrophoresis. Protein expression of (**A**) phosphorylated-eIF2α, (**B**) ATF3, (**C**) active/cleaved ATF6, and (**D**) phosphorylated-IRE1α was detected using the specific antibodies. β-Actin expression was used as a loading control. The arrow indicates the target band. After being normalized to the loading control, the target bands were quantified by densitometry using the ImageJ software (version 1.51, National Institutes of Health, Bethesda, MD, USA). The relative protein expression level at pH 7.4 of each EC type was set as 1.0-fold; (**E**) HUVEC, HPAEC, and HMVEC-L cells were treated at pH 8.4, 7.4, or 6.4 for 5 h. Total RNA was isolated and cDNA was synthesized. Unspliced and spliced human XBP-1 mRNA isoforms were examined by reverse transcription polymerase chain reaction (RT-PCR), as described in “Materials and Methods”. The results shown are representative of at least two biological repeats.

To assess the effects of acidosis on ER stress/UPR in primary EC cultures, we used human umbilical vein endothelial cells (HUVEC), human pulmonary artery endothelial cells (HPAEC), and human lung microvascular endothelial cells (HMVEC-L) as model systems. ECs were treated with physiological pH 7.4 or acidic pH 6.4, and the genes involved in the ER stress/UPR pathways (PERK, ATF6, and IRE1α) were examined. Previous studies showed that GPR4 exhibits a low level of activation at pH 7.4 and is fully activated at pH 6.4 [1,2,21]. As shown in Figure 1, acidic pH treatment of HUVEC, HPAEC, and HMVEC-L caused the activation of the PERK pathway, as demonstrated by the increased phosphorylation of eIF2α and increased expression of the downstream gene *ATF3* (Figure 1A,B). Moreover, acidic pH treatment also activated the ATF6 pathway and the IRE1α pathway, as demonstrated by the increased expression of the active/cleaved form of ATF6 (50 kDa) as well as the increased phosphorylation of IRE1α and increased expression of the spliced isoform of XBP-1 mRNA (Figure 1C–E). Together, these results demonstrate that acidosis activates all three arms of the ER stress/UPR pathways (PERK, ATF6, and IRE1α) in primary human ECs.

### 2.2. Overexpression of GPR4, but Not the Signaling Defective GPR4 Mutant, Augments the ER Stress Response Induced by Acidosis in HUVEC

**Figure 2:**
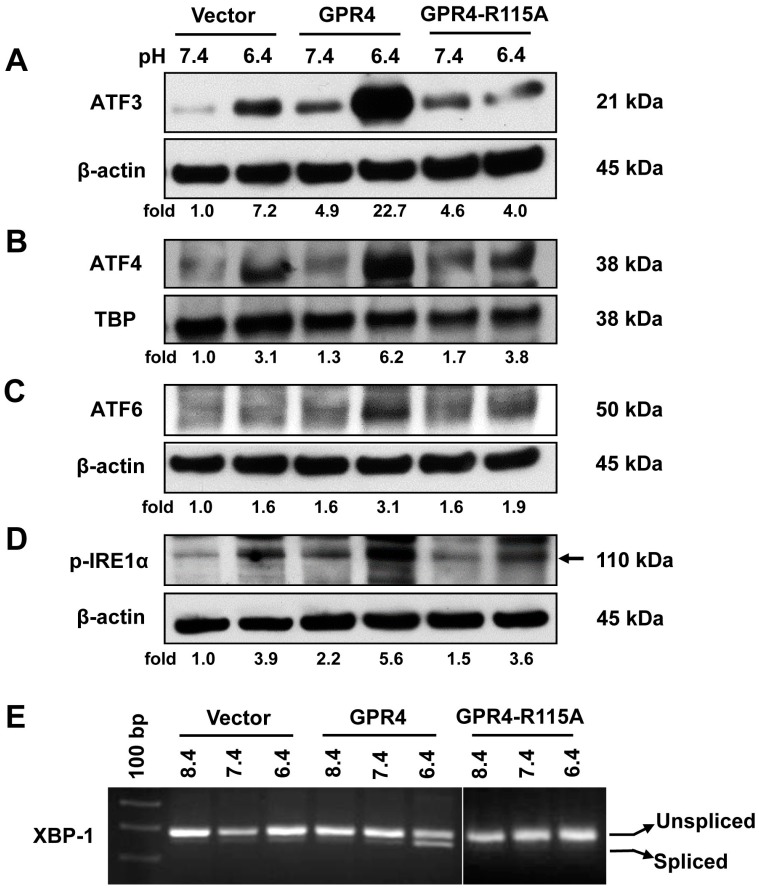
Acidosis-induced ER stress response is augmented by overexpression of G protein-coupled receptor 4 (GPR4), but not the signaling defective GPR4 mutant, in HUVEC. (**A**–**D**) HUVECs transduced with the control vector (Vector), GPR4 expression construct (GPR4), or GPR4 R115A mutant expression vector (GPR4 R115A) were treated in EGM-2/HEM buffered media at physiological (pH 7.4) or acidic (pH 6.4) conditions for 0.5–1 h (p-IRE1α) or 5 h (ATF3, ATF4, and ATF6). Cell lysates were then collected and separated by electrophoresis. Protein expression of (**A**) ATF3, (**B**) ATF4, (**C**) active/cleaved ATF6, and (**D**) phosphorylated-IRE1α was detected using the specific antibodies. β-Actin or TATA-binding protein (TBP) expression was used as a loading control. The arrow indicates the target band. After being normalized to the loading control, the target bands were quantified by densitometry using the ImageJ software (version 1.51, National Institutes of Health, Bethesda, USA). The relative protein expression level at pH 7.4 of HUVEC/Vector was set as 1.0-fold. (**E**) HUVECs, as used in (**A**–**D**), were treated at pH 8.4, 7.4, or 6.4 for 5 h. Total RNA was isolated and cDNA was synthesized. Spliced and unspliced XBP-1 mRNA isoforms were examined by RT-PCR. The results shown are representative of at least two biological repeats.

It has previously been shown that the proton-sensing receptor GPR4 is a functional acid sensor in ECs [1,2,20,21]. To investigate the role of GPR4 in the acidosis-induced ER stress response, we stably transduced HUVEC with the MSCV-IRES-GFP (murine stem cell virus-internal ribosomal entry site-green fluorescent protein) retroviral vector carrying the wild-type *GPR4* gene (HUVEC/GPR4), the signaling defective GPR4-R115A mutant (HUVEC/GPR4-R115A), or the empty vector (HUVEC/Vector), as previously described [1]. HUVEC/Vector cells have endogenous GPR4 expression, whereas HUVEC/GPR4 cells and HUVEC/GPR4-R115A cells have overexpression of wild-type GPR4 and mutated GPR4, respectively [1]. HUVECs were treated with pH 7.4 and pH 6.4. The results showed that acidic pH treatment increased the protein expression of ATF3, ATF4, and active/cleaved ATF6 to a higher level in HUVEC/GPR4 cells when compared to HUVEC/Vector cells (Figure 2A–C). However, overexpression of the GPR4-R115A mutant did not further increase the expression of these genes. Similar results were observed with regard to acidosis/GPR4-induced IRE1α phosphorylation and XBP-1 mRNA splicing (Figure 2D,E). These results demonstrate that overexpression of GPR4, but not the signaling defective GPR4-R115A mutant, further potentiates acidosis-induced ER stress response in ECs.

### 2.3. Knocking Down GPR4 by shRNA Attenuates the ER Stress Response Induced by Acidosis in HUVEC

**Figure 3:**
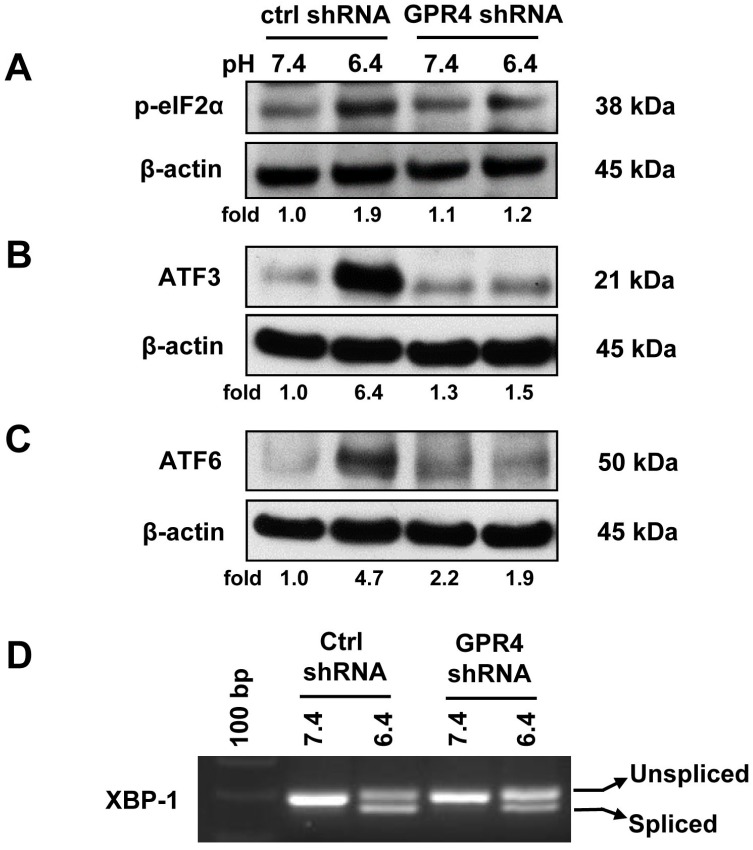
Acidosis-induced ER stress response is alleviated by knocking down GPR4 expression in HUVEC. HUVECs transduced with the control shRNA (ctrl shRNA) or GPR4 shRNA (GPR4 shRNA) were treated in EGM-2/HEM buffered media at physiological (pH 7.4) or acidic (pH 6.4) conditions for 0.5–1 h (p-eIF2α), 1 h (XBP-1), or 5 h (ATF3 and ATF6). (**A**–**C**) Cell lysates were collected and separated by electrophoresis. Protein expression of (**A**) phosphorylated-eIF2α, (**B**) ATF3, and (**C**) active/cleaved ATF6 was detected using the specific antibodies. β-Actin expression was used as a loading control. After being normalized to the loading control, the target bands were quantified by densitometry using the ImageJ software (version 1.51, National Institutes of Health, Bethesda, USA). The relative protein expression level at pH 7.4 of HUVEC/Control-shRNA was set as 1.0-fold. (**D**) Total RNA was isolated from the cells and cDNA was synthesized. Spliced and unspliced XBP-1 isoforms were examined by RT-PCR. The results shown are representative of two or more biological repeats.

To determine the dependency of GPR4 in association with an acidosis-induced ER stress response, GPR4 mRNA expression was stably knocked down by ~90% using a GPR4-specific shRNA in HUVEC (HUVEC/GPR4-shRNA), as previously described [1]. HUVEC/Control-shRNA and HUVEC/GPR4-shRNA cells were treated in physiological or acidic pH media. Compared to HUVEC/Control-shRNA cells, the expression of ER stress/UPR marker genes, such as phosphorylated-eIF2α, ATF3, active/cleaved ATF6, and spliced XBP-1 mRNA, was reduced in HUVEC/GPR4-shRNA cells in response to acidic pH (Figure 3A–D). The results indicate that GPR4 expression is required, at least in part, for acidosis-induced ER stress response in ECs.

### 2.4. Blockade of GPR4 Activity by a Small Molecule Inhibitor Diminishes the ER Stress Response Induced by Acidosis in HUVEC

**Figure 4:**
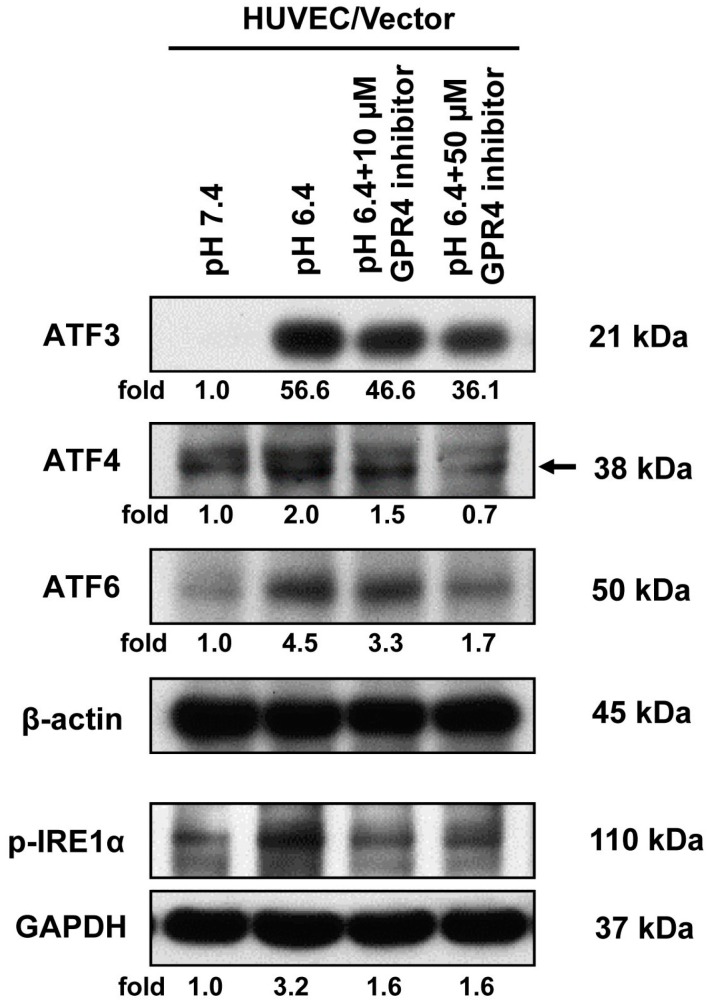
Acidosis-induced ER stress response is attenuated by a GPR4 small molecule inhibitor in HUVEC. HUVEC/Vector cells were treated for 0.5–1 h (p-IRE1α) or 5 h (ATF3, ATF4 and ATF6) with EGM-2/HEM pH 7.4 and 6.4 media or with pH 6.4 media containing 10 and 50 µM of the GPR4 inhibitor, for which one-hour pretreatment in EGM-2 medium with the same concentrations of GPR4 inhibitor was performed. Cell lysates were then collected and separated by electrophoresis. Protein expression of ATF3, ATF4, active/cleaved ATF6, and phosphorylated-IRE1α was detected using the specific antibodies. β-actin or GAPDH expression was used as a loading control. The arrow indicates the target band. After being normalized to the loading control, the target bands were quantified by densitometry using the ImageJ software (version 1.51, National Institutes of Health, Bethesda, MD, USA). The relative protein expression level at pH 7.4 of HUVEC/Vector was set as 1.0-fold. The results shown are representative of two or more biological repeats.

Previously, a GPR4 inhibitor, 2-Ethyl-3-(4-((*E*)-3-(4-isopropyl-piperazin-1-yl)-propenyl)-benzyl)-5,7-dimethyl-3H-imidazo(4,5-b)pyridine (EIDIP), has been demonstrated to lessen acidosis-induced EC inflammation in HUVEC/Vector and HUVEC/GPR4 [2]. Other similar GPR4 inhibitors have also been shown to reduce acidosis-induced EC inflammation and promote survival in a mouse myocardial infarction model [21,23]. We aimed to use the GPR4 inhibitor (EIDIP) to study the acidosis-induced endothelial ER stress/UPR effects. HUVEC/Vector cells were treated at physiological pH and acidic pH with different concentrations of the GPR4 inhibitor (10 and 50 µM). The increase in ER stress protein expression in response to acidosis has been attenuated in correlation with increasing GPR4 inhibitor concentration, as displayed by Western blotting for specific UPR markers in vascular ECs, including ATF3, ATF4, active/cleaved ATF6, and phosphorylated IRE1α (Figure 4). Together, these results show that the blockade of GPR4 activity by its inhibitor reduces acidosis-induced ER stress response in ECs, further substantiating the role of GPR4 in this process.

### 2.5. GPR4 Modulates the mRNA Expression of ER Stress Response Genes Induced by Acidic pH in HUVEC

**Figure 5:**
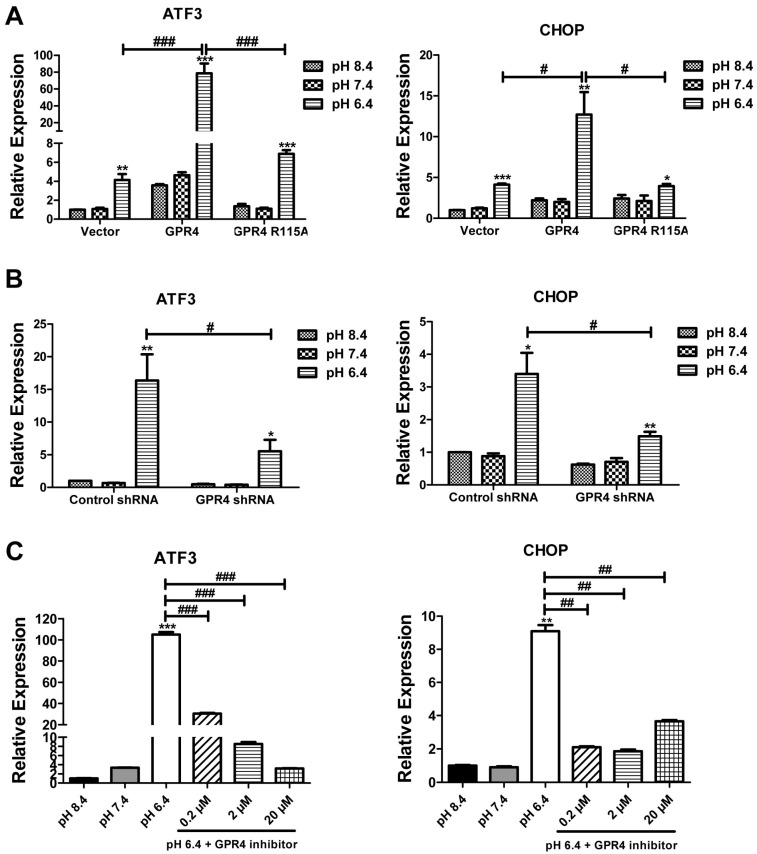
GPR4 modulates the ER stress response genes induced by acidic pH at the mRNA level in HUVEC. (**A**,**B**) HUVEC transduced with the control vector (Vector), GPR4 expression construct (GPR4), GPR4 R115A mutant expression vector (GPR4 R115A), control shRNA (control shRNA), or GPR4 shRNA expression vector (GPR4 shRNA) were treated in EGM-2/HEM buffered media at basic (pH 8.4), physiological (pH 7.4), or acidic (pH 6.4) conditions for 5 h. (**C**) HUVEC/GPR4 cells were treated for 5 h with EGM-2/HEM pH 8.4, 7.4, or 6.4 media or with pH 6.4 media containing 0.2, 2, and 20 µM of the GPR4 inhibitor, for which one-hour pretreatment in EGM-2 medium with the same concentrations of GPR4 inhibitor was performed. Total RNA was isolated, and cDNA was synthesized. Real-time qRT-PCR was performed to quantify the mRNA level of ATF3 and CHOP. *C*_t_ values were normalized to the housekeeping gene β-actin (ACTB). The expression level of the target genes in (**A**) HUVEC/Vector, (**B**) HUVEC/control shRNA or (**C**) HUVEC/GPR4 cells treated with pH 8.4 was set as 1. Error bars indicate the mean ± SEM. *, *p* < 0.05; **, *p* < 0.01; ***, *p* < 0.001; compared with corresponding pH 8.4 groups. #, *p* < 0.05; ##, *p* < 0.01; ###, *p* < 0.001; comparing the indicated pairs of data. The results shown are the average of at least two biological repeats.

To further investigate the role of GPR4 in the ER stress response, the mRNA of UPR downstream markers was assessed. HUVECs transduced with a control vector (Vector), GPR4 (GPR4), GPR4 R115A mutant (GPR4 R115A), control shRNA (control shRNA), and GPR4 shRNA expression vector (GPR4 shRNA) were treated in EGM-2/HEM buffered media at basic (pH 8.4), physiological (pH 7.4), or acidic (pH 6.4) conditions. In Figure 5A, the mRNA expressions of ATF3 (a downstream marker of the PERK/eIF2α pathway) and CHOP (a downstream effector of the ATF6 and PERK/eIF2α pathways) were analyzed by real-time qRT-PCR. Acidosis increased the expression of ATF3 and CHOP mRNA by ~5-fold compared to basic treatment conditions in HUVEC/Vector cells that have endogenous GPR4 expression. With the overexpression of GPR4 in HUVEC/GPR4 cells, acidosis significantly further increased the expression of ATF3 and CHOP mRNA. In contrast, overexpression of the GPR4 mutant (GPR4 R115A) did not increase ATF3 or CHOP expression to the same extent as the wild-type GPR4 overexpression (Figure 5A). With the knockdown of GPR4 (GPR4-shRNA), acidosis-stimulated ATF3 and CHOP mRNA expression was significantly reduced in HUVEC compared to the control shRNA (Figure 5B). Furthermore, HUVEC/GPR4 cells were treated with acidic pH in the presence or absence of the GPR4 small molecule inhibitor, and the mRNA expressions of ATF3 and CHOP were analyzed. The results showed that the GPR4 inhibitor significantly inhibited acidosis-induced ATF3 and CHOP mRNA expression in ECs (Figure 5C).

### 2.6. GPR4 Modulates Hypercapnic Acidosis-Induced ER Stress Gene Expression in HUVEC

**Figure 6:**
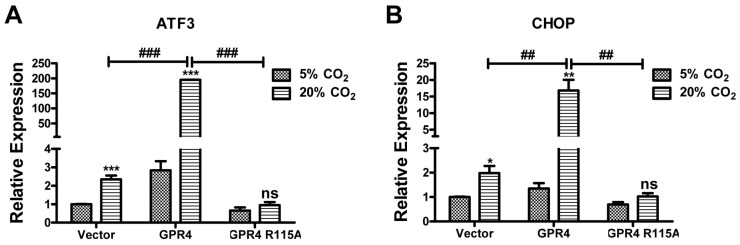
GPR4 is involved in modulating the ER stress response induced by hypercapnic acidosis in HUVEC. HUVECs transduced with the control vector (Vector), GPR4 expression construct (GPR4), or GPR4 R115A mutant expression vector (GPR4 R115A) were treated for 5 h with EGM-2 media buffered with 5% CO_2_ or 20% CO_2_. Total RNA was isolated and cDNA was synthesized. Real-time qRT-PCR was performed to quantify the mRNA level of (**A**) ATF3 and (**B**) CHOP. *C*_t_ values were normalized to the housekeeping gene β-actin (ACTB). The expression level of the target genes in HUVEC/Vector cells treated with 5% CO_2_-buffered EGM-2 medium was set as 1. Error bars indicate the mean ± SEM. *, *p* < 0.05; **, *p* < 0.01; ***, *p* < 0.001; ns, not significant (*p* > 0.05); compared with corresponding “5% CO_2_” groups. #, *p* < 0.05; ##, *p* < 0.01; ###, *p* < 0.001; comparing the indicated pairs of data. The results shown are the average of at least two biological repeats.

In addition to isocapnic acidosis, hypercapnic acidosis is another type of acidosis that is caused by an increased concentration of carbon dioxide (CO_2_) in the body and is commonly associated with respiratory disorders [1,2,24]. To simulate hypercapnic acidosis, HUVEC/Vector, HUVEC/GPR4, and HUVEC/GPR4-R115A cells were treated with EGM-2 medium under 5% CO_2_ (~pH 7.4) and 20% CO_2_ (~pH 6.4) for 5 h. Similar to isocapnic acidosis, hypercapnic acidosis stimulated the mRNA expression of downstream ER stress/UPR marker genes *ATF3* and *CHOP* in HUVEC/Vector cells. The expression of these genes was further augmented by the overexpression of wild-type GPR4 but not the GPR4-R115A mutant (Figure 6). These results show that hypercapnic acidosis also stimulates ER stress response through GPR4 in ECs.

## 3. Discussion

Acidosis is a noxious stressor associated with many pathological conditions such as cancer, ischemia, and inflammation [2,3,4,5,6,7,8,18]. Glycolytic cell metabolism, hypoxia, and defective blood perfusion are the main causes of excessive proton production and accumulation in the affected tissue, resulting in an acidotic tissue microenvironment [3,6,7,8]. Acidosis has been shown to induce cell apoptosis, inhibit cell proliferation, and modulate inflammatory responses [2,3,4,5,6,7,8]. In tumors, acidosis can act as a selective pressure to drive cancer cell somatic evolution, against which autophagy is a protective mechanism for the development of acidosis-resistant cancer cells [25,26]. In ischemic tissues, acidosis aggravates ischemia-related tissue injury. For example, brain tissue pH can drop to as low as 6.0–6.5 during a stroke, and acidotic stress has been shown to increase neural cell apoptosis [6,7,27].

**Figure 7:**
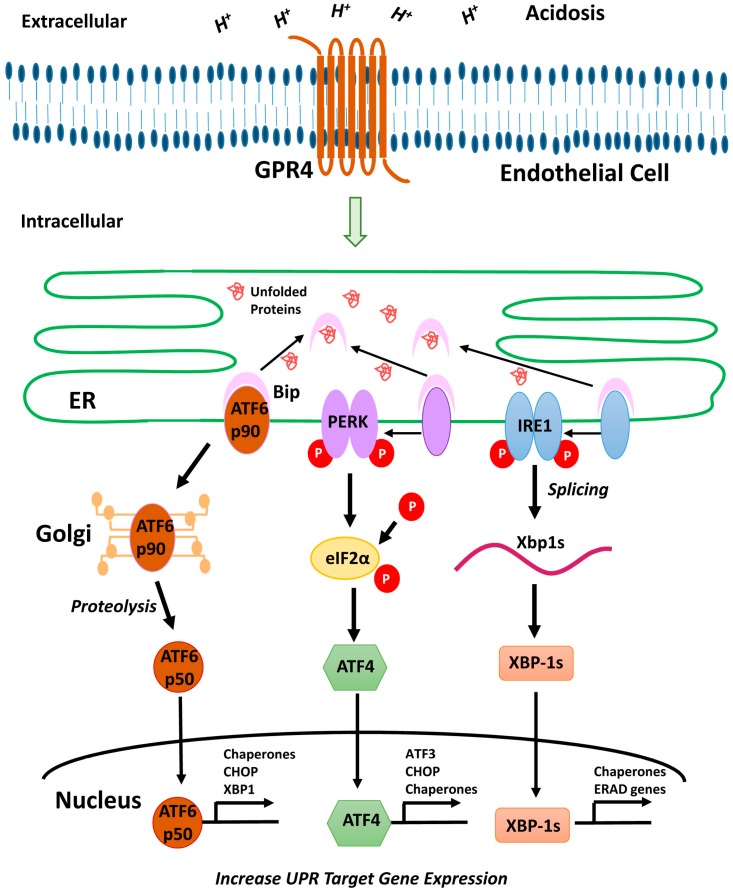
A model depicting the acidosis/GPR4-induced ER stress response in vascular ECs. Acidic extracellular pH activates the proton-sensing receptor GPR4 and stimulates all three arms (ATF6, PERK, and IRE1) of the ER stress/UPR pathways in ECs. “P” denotes the phosphate group of a protein.

Previous studies demonstrate that acidosis can induce ER stress response in several cell types. Aoyama et al. reported that acidosis induces ER stress in astrocytes and causes astrocyte death [9]. Tang et al. performed microarray analyses and reported that lactic acidosis increases the expression of several UPR genes, including *CHOP*, *XBP-1*, and *ATF3*, in cancer cells [11]. Johno et al. reported that acidic stress elicits ER stress response in mesothelial cells [10]. Visioli et al. reported that acidosis increases ER stress in ECs, and the expression of UPR markers is significantly higher in tumor-associated ECs compared to primary human dermal microvascular ECs [12]. Although acidosis has been shown to induce ER stress, molecular mechanisms by which cells sense acidosis to trigger ER stress/UPR were unknown. We previously performed microarray analyses to identify acidosis-responsive genes in vascular ECs [2]. Our results showed that acidosis increases the expression of several ER stress genes, such as *CHOP* and *ATF3*, as well as numerous inflammatory genes in ECs [2]. Overexpression of the proton-sensing GPR4 receptor further augments the expression of these genes in response to acidosis in ECs [2]. In the current study, we aimed to comprehensively investigate the role of GPR4 in the acidosis-induced ER stress response in ECs. Our results showed that acidosis increases the expression of the UPR marker genes involved in all three arms of the ER stress pathways in vascular ECs that have endogenous GPR4 expression (Figure 1). Overexpression of GPR4, but not a signaling-defective mutant, further augments acidosis-induced UPR marker gene expression in ECs (Figure 2, Figure 5A and Figure 6). Conversely, knockdown of GPR4 by shRNA and inhibition of GPR4 by small molecule inhibitors diminishes acidosis-induced ER stress gene expression (Figure 3, Figure 4 and Figure 5B,C). Therefore, by using both gain- and loss-of-function approaches, our study clearly demonstrates that GPR4 is a functional pH sensor and is important for ECs to sense acidosis and elicit an ER stress response (Figure 7). While a mild ER stress is protective, a severe prolonged ER stress is detrimental to cells [13,14,15]. As discussed above, acidosis-induced ER stress has been shown to cause astrocyte death [9]; however, acidosis-induced ER stress and GRP78 expression is protective for ECs in response to the Sunitinib drug treatment [12]. Our preliminary results showed that acidotic treatment with pH 6.4 for 24 h modestly increases cell death in HUVEC when compared to pH 7.4 (~3% vs. ~5% dead cells). Overexpression of GPR4, but not the GPR4-R115A mutant, slightly further increases, and knockdown of GPR4 moderately reduces, HUVEC cell death under pH 6.4 (Figure S1). Further research is warranted to define the role of GPR4 in EC viability under acidotic conditions.

GPR4, GPR65 (TDAG8), GPR68 (OGR1), and possibly GPR132 (G2A), constitute the proton-sensing G protein-coupled receptors (GPCRs) [4,5,18,19]. These receptors can be activated by acidic extracellular pH through the protonation of several histidine residues [19]. The activation of GPR4 by acidic pH has been shown to increase the expression of inflammatory genes (chemokines, cytokines, adhesion molecules, etc.) in ECs [1,2,21]. Another study shows that GPR4 activation by acidosis stimulates cyclic adenosine monophosphate/protein kinase A (cAMP/PKA) signaling, which leads to the upregulation of the receptor activator of nuclear factor kappa-B ligand (RANKL) cytokine expression in osteoblasts [28]. Our recent publication highlights GPR4 as being pro-inflammatory within inflamed intestinal tissues, as the deletion of GPR4 reduces intestinal inflammation in an acute experimental colitis model [22]. The expression of E-selectin and vascular cell adhesion molecule-1 (VCAM-1) proteins in ECs was decreased in the inflamed gut mucosa of GPR4-deficient mice compared to wild-type (WT) mice [22]. Interestingly, ER stress pathways have been shown to stimulate inflammation [15]. Further research will need to delineate any potential connections between acidosis/GPR4 induced ER stress response and inflammation in ECs. Whereas GPR4 is predominately expressed in ECs and macrophages [1,2,22], acidosis can induce ER stress in multiple cell types as discussed above [2,9,10,11,12]. It remains to be determined if other pH-sensing receptors, such as GPR65 and GPR68, can also elicit ER stress in response to acidosis in the cell types in which the receptors are expressed. In addition to pH-sensing GPCRs, it should be noted that several types of ion channels, such as acid-sensing ion channels (ASICs) and transient receptor potential V (TRPV) cation channels, can also function as acid sensors and play a role in acidosis-induced stress responses [17].

Modulation of the ER stress/UPR pathways has been exploited for disease treatment [29]. Bortezomib, an ER stress-inducing agent, can induce cancer cell apoptosis and has been approved by the FDA for treating multiple myeloma and mantle cell lymphoma [30]. On the other hand, inhibition of ER stress has been evaluated as a potential approach for the treatment of inflammation, cardiovascular disease, metabolic disease, and neurodegenerative disease [31]. In the current study, our results demonstrate that treatment with GPR4 small molecule inhibitor or shRNA inhibitor attenuates acidosis-induced ER stress in ECs (Figure 3, Figure 4 and Figure 5). Previously, we, and others, have shown that GPR4 inhibitors diminishes acidosis-induced inflammatory gene expression in ECs [1,2,21]. A recent study further demonstrates that treatment with a GPR4 inhibitor prolongs animal survival in a myocardial infarction mouse model [23]. Taken together, these studies suggest that inhibition of GPR4 can alleviate acidosis-induced ER stress and inflammation in ECs and be explored as a novel approach for the treatment of inflammatory and ischemia-related disorders.

## 4. Materials and Methods

### 4.1. Chemicals and Reagents

Polyclonal antibodies for phosphorylated eIF2α (Ser51) and TBP (TATA-binding protein), as well as monoclonal antibodies for β-actin (clone 13E5) and GAPDH (clone 14C10), were purchased from Cell Signaling Technology (Danvers, MA, USA). Polyclonal antibodies for ATF6α and ATF4 (CREB2) were from Santa Cruz Biotechnology (Dallas, TX, USA). The antibody for ATF3 was purchased from GeneTex (Irvine, CA, USA), and the antibody for phosphorylated IRE1α (Ser724) was purchased from Thermo Fisher Scientific (Waltham, MA, USA). 4-(2-hydroxyethyl)-1-piperazineethanesulfonic acid (HEPES), *N*-(2-hydroxyethyl)-piperazine-*N*′-3-propanesulfonic acid (EPPS), 2-(4-morpholino)-ethanesulfonic acid (MES), and the protease inhibitor cocktail were from Sigma-Aldrich (St Louis, MO, USA) and Thermo Fisher Scientific (Waltham, MA, USA). The GPR4 inhibitor, 2-Ethyl-3-(4-((*E*)-3-(4-isopropyl-piperazin-1-yl)-propenyl)-benzyl)-5,7-dimethyl-3*H*-imidazo(4,5-b)pyridine, was purchased from Dalton Pharma Services (Toronto, ON, Canada).

### 4.2. Cell Culture and Retroviral Transduction

A humidified tissue culture incubator was filled with 5% CO_2_ and 95% air and set at 37 °C for cell culture. Primary human umbilical vein endothelial cells (HUVEC), human pulmonary artery endothelial cells (HPAEC), and human lung microvascular endothelial cells (HMVEC-L) were purchased from Lonza (Walkersville, MD, USA). HUVEC and HPAEC were cultured in endothelial cell growth medium 2 (EGM-2), while HMVEC-L was grown in EGM-2-MV medium (Lonza, Walkersville, MD, USA). HUVECs stably expressing the MSCV-IRES-GFP (HUVEC/Vector), MSCV-huGPR4-IRES-GFP (HUVEC/GPR4), MSCV-huGPR4 R115A-IRES-GFP (HUVEC/GPR4-R115A), Flink-control shRNA (HUVEC/Control-shRNA), or Flink-huGPR4 shRNA (HUVEC/GPR4-shRNA) were obtained by cell transduction with construct-containing retroviral/lentiviral particles and then by fluorescence-activated cell sorting (FACS), based on green fluorescence signals as previously described [1].

### 4.3. Isocapnic and Hypercapnic pH Treatment

To prepare isocapnic pH media, a previously described procedure was followed [1,2]. Briefly, EGM-2 or EGM-2-MV media were first buffered with 7.5 mM HEPES, 7.5 mM EPPS, and 7.5 mM MES (abbreviated as HEM), and the pH of the media was then measured with an electronic pH meter (Thermo Fisher Scientific) using NaOH or HCl to adjust to the desired pH. To prepare the hypercapnic pH media, regular EGM-2 medium was incubated overnight in humidified tissue culture incubators with 5% CO_2_ or 20% CO_2_. After the overnight pre-treatment, the pH of each medium was measured to be around 7.4 and 6.4, respectively. Prior to the pH treatment, human ECs were cultured in 6-cm plates to reach 50%–90% confluency. To perform isocapnic pH treatment, the ECs were incubated for the desired length of time in the EGM-2/HEM or EGM-2-MV/HEM media at varying pH conditions in a regular tissue culture incubator with 5% CO_2_. To perform hypercapnic pH treatment, the ECs were treated with CO_2_-buffered EGM-2 media for the desired length of time in tissue culture incubators filled with the same concentration of CO_2_ as used for pre-treatment (5% CO_2_ or 20% CO_2_). When the GPR4 inhibitor was used, the ECs were pretreated with regular growth medium containing indicated concentrations of the GPR4 inhibitor for 1 h and then treated for 5 h with HEM-buffered growth medium at varying pH conditions, which contained the same concentrations of GPR4 inhibitor as used in pretreatment.

### 4.4. Western Blotting

For whole cell lysate preparation, after the indicated length of pH treatment, ECs were lysed in ice-cold radioimmune precipitation assay (RIPA) buffer followed by high-speed centrifuge, as previously described [1]. Nuclear fraction of the cell lysate was obtained by using the Nuclear Extraction Kit (Millipore Sigma, Billerica, MA, USA). Protein concentration of the cell lysate was determined by the DC™ Protein Assay (Bio-Rad, Hercules, CA, USA). The desired amount of cell lysates were then separated by SDS-PAGE and transferred onto nitrocellulose membrane (GE Healthcare, Chicago, IL, USA). The expression of phosphorylated-eIF2α, phosphorylated-IRE1a, ATF4, ATF6, ATF3, TBP, β-actin, and GAPDH was analyzed by Western blotting with corresponding primary antibodies and the horseradish peroxidase (HRP)-conjugated secondary antibodies (Santa Cruz Biotechnology, Dallas, TX, USA). Chemiluminescence signals were detected using the Amersham ECL Advance Western blotting detection kit, following the manufacturer’s instructions (GE Healthcare). Relative protein expression levels of the target bands were quantified by densitometry using the ImageJ software (available on: https://imagej.nih.gov/ij/).

### 4.5. Reverse Transcription Polymerase Chain Reaction (RT-PCR)

Total RNA was isolated from the ECs using the Qiagen RNeasy Mini Kit (Vilencia, CA, USA). Reverse transcription (RT) and polymerase chain reaction (PCR) reagents were purchased from Thermo Fisher Scientific. PCR was performed with a pair of primers that can specifically amplify both the spliced and unspliced human XBP-1 isoforms [32]. PCR was initiated at 95 °C for 2 min, followed by 35 cycles of 94 °C for 30 s, 58 °C for 30 s, and 72 °C for 1 min. Electrophoresis of the PCR products was then performed using a 2% agarose gel and the DNA was stained with ethidium bromide (Millipore Sigma, Billerica, MA, USA). The gel images were captured using the UVP Benchtop UV transilluminator (UVP BioDocIt Imaging System, Upland, CA, USA).

### 4.6. Real-Time qRT-PCR

Real-time qPCR reagents were purchased from Thermo Fisher Scientific. The primers specific for the target genes were the TaqMan Gene Expression Assays from Thermo Fisher Scientific, *ATF3* (Hs00231069_ml), and *DDIT3/CHOP* (Hs01090850_ml). *ACTB/β-actin* (Hs99999903_m1) was used as the internal control. Real-time qPCR was performed in duplicate and the data were acquired and analyzed using the ABI 7300-HT real-time PCR thermocycler (Thermo Fisher Scientific). PCR was initiated at 50 °C for 2 min and 95 °C for 10 min, followed by 40 cycles of 95 °C for 15 s and 60 °C for 1 min. The fold of the gene expression changes was calculated using the 2^−∆∆*C*t^ method [33].

### 4.7. Statistical Analysis

The data were analyzed using GraphPad Prism 5 software (version 5.01, GraphPad Software, Inc., San Diego, CA, USA). The differences between two experimental groups were compared using the unpaired *t*-test. *p* < 0.05 was considered statistically significant.

## 5. Conclusions

In this study we have investigated the role of the proton-sensing GPR4 receptor in acidosis-induced ER stress response in vascular ECs. Our results show that acidosis activates all three arms (PERK, ATF6, and IRE1) of the ER stress/UPR pathways in primary EC cultures. Secondly, GPR4 is a functional pH sensor that is important for the regulation of acidosis-induced ER stress in ECs. Thirdly, a GPR4 small molecule inhibitor can attenuate an endothelial ER stress response triggered by acidosis. These results suggest that the modulation of GPR4 by its agonists and antagonists can stimulate and inhibit ER stress/UPR in vascular ECs, respectively, and be exploited for potential therapeutic applications.

## Supplementary Materials

Supplementary materials can be found at www.mdpi.com/1422-0067/18/2/278/s1.

## Abbreviations

ATF, activating transcription factor; cAMP, cyclic adenosine monophosphate; CHOP, C/EBP homologous protein; EC, endothelial cell; EGM-2, endothelial cell growth medium 2; EIDIP, 2-Ethyl-3-{4-[(E)-3-(4-isopropyl-piperazin-1-yl)-propenyl]-benzyl}-5,7-dimethyl-3H-imidazo[4,5-b]pyridine; eIF2α, eukaryotic initiation factor 2α; EPPS, *N*-(2-hydroxyethyl)-piperazine-*N*′-3-propanesulfonic acid; ER, endoplasmic reticulum; ERAD, endoplasmic reticulum associated protein degradation; GAPDH, glyceraldehyde 3-phosphate dehydrogenase; GPR4, G protein-coupled receptor 4; HEPES, 4-(2-hydroxyethyl)-1-piperazineethanesulfonic acid; HMVEC-L, human lung microvascular endothelial cell; HPAEC, human pulmonary artery endothelial cell; HUVEC, human umbilical vein endothelial cell; IRE1, inositol-requiring enzyme 1; MES, 2-(4-morpholino)-ethanesulfonic acid; MSCV-IRES-GFP, murine stem cell virus-internal ribosomal entry site-green fluorescent protein; PERK, protein kinase R-like ER kinase; PKA, protein kinase A; RT-PCR, reverse transcription polymerase chain reaction; shRNA, short hairpin RNA; TBP, TATA-binding protein; UPR, unfolded protein response; VCAM-1, vascular cell adhesion molecule-1; XBP-1, X box-binding protein 1.
