# Supplementary material for: Acidosis Activates Endoplasmic Reticulum Stress Pathways through GPR4 in Human Vascular Endothelial Cells
Source: Int J Mol Sci. 2017 Jan 27;18(2):278. doi: 10.3390/ijms18020278 (PMC5343814; doi:10.3390/ijms18020278)
Supplement: Supplementary file 1 [file ijms-18-00278-s001.docx]

Supplementary Materials: Acidosis Activates Endoplasmic Reticulum Stress Pathways through GPR4 in Human Vascular Endothelial Cells

Lixue Dong, Elizabeth A. Krewson and Li V. Yang


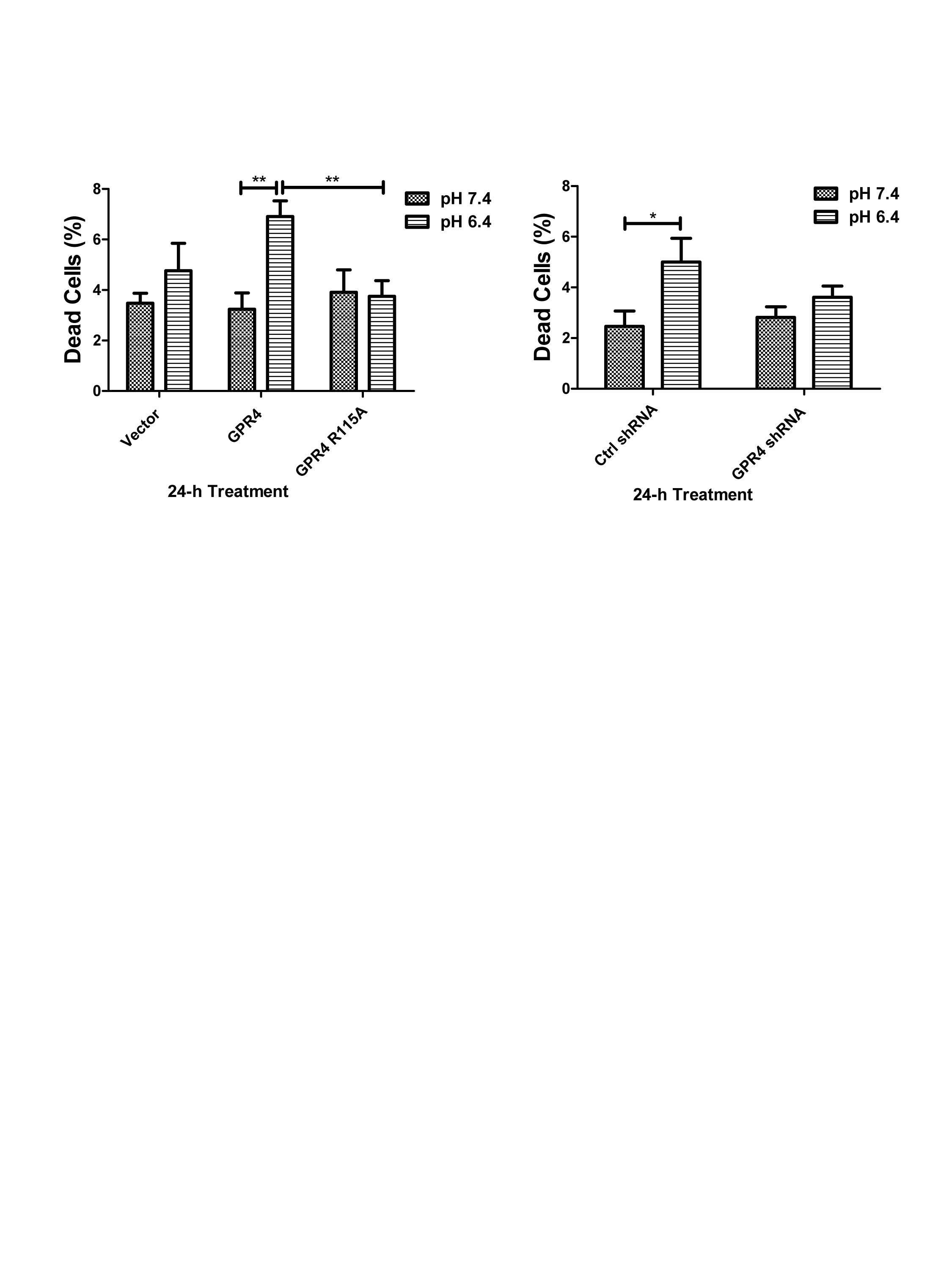


**Figure S1.** Analysis of cell death under acidotic stress. HUVEC/Vector, GPR4, GPR4-R115A, Control-shRNA, and GPR4-shRNA cells were cultured in a 96-well plate to reach ~50% confluency. The cells were pretreated with EGM-2/HEM buffered media (pH 7.4) overnight, followed by a 24-h treatment with EGM-2/HEM media at pH 7.4 or 6.4. Post treatment, dead cells were detected using ethidium homodimer-1 (Thermo Fisher Scientific, Waltham, MA, USA), which specifically stains the nuclear DNA of dead or dying cells. Pictures were taken using an inverted fluorescence microscope to capture the dead cell count and the total cell count. The percentage of dead cells was calculated as the ratio of the dead cell count to the total cell count. Error bars indicate the mean ± SEM (*n* = 6–8). *, *p* < 0.05; **, *p* < 0.01.
